# Supplementary material for: Computer-Assisted Intraoperative Navigation in Pediatric Head and Neck Surgical Oncology: A Single-Center Case Series and Scoping Review of the Literature
Source: Cancers (Basel). 2026 Jan 1;18(1):154. doi: 10.3390/cancers18010154 (PMC12785002; doi:10.3390/cancers18010154)
Supplement: Supplementary file 1 [file cancers-18-00154-s001.zip › Intraoperative navigation Search strategy.pdf]

Intraoperative Navigation Search Strategy:

PUBMED:

("Pediatrics"[Mesh] OR "Child"[Mesh] OR pediatric* OR paediatric* OR child* OR adolescen* OR teen* OR infant*)

AND ("Neuronavigation"[Mesh] OR "Surgical Navigation Systems"[Mesh] OR "intraoperative navigat*" OR neuronavigat* OR "surgical navigat*" OR "stereotactic navigat*" OR "stealthstation" OR CranialMap OR NAV3i OR Kick OR Stryker OR BrainLAB OR Medtronic)

AND ("Neoplasms"[Mesh] OR cancer* OR tumor* OR tumour* OR osteosarcoma OR rhabdomyosarcoma OR sarcoma OR lymphoma OR carcinoma OR histiocytosis OR Langerhan's)

AND ("Head"[Mesh] OR "Neck"[Mesh] OR "Otolaryngology"[Mesh] OR "Larynx"[Mesh] OR "Salivary Glands"[Mesh] OR "Skull"[Mesh] OR "Cervical Vertebrae"[Mesh] OR "Paranasal Sinuses"[Mesh] OR "Thyroid Gland"[Mesh] OR head OR heads OR neck* OR skull OR cranium OR cranial OR Craniofacial OR mandib* OR maxillary OR orbital OR paranasal OR sinonasal OR clival OR clivus OR thyroid OR laryngeal OR larynx OR salivary)

EMBASE:

('**pediatrics**'/exp OR '**child**'/exp OR '**pediatric***' OR '**paediatric***' OR '**child***' OR '**adolescen***' OR '**teen***' OR '**infant***') AND ('**neuronavigation**'/exp OR '**surgical navigation system**'/exp OR '**intraoperative navigat***' OR '**neuronavigat***' OR '**surgical navigat***' OR '**stereotactic navigat***' OR '**stealthstation**' OR '**cranialmap**' OR '**nav3i**' OR '**kick**' OR '**stryker**' OR '**brainlab**' OR '**medtronic**') AND ('**neoplasm**'/exp OR '**cancer***' OR '**tumor***' OR '**tumour***' OR '**osteosarcoma**' OR '**rhabdomyosarcoma**' OR '**sarcoma**' OR '**lymphoma**' OR '**carcinoma**' OR '**histiocytosis**' OR '**langerhan`s**') AND ('**head**'/exp OR '**neck**'/exp OR '**otorhinolaryngology**'/exp OR '**larynx**'/exp OR '**salivary gland**'/exp OR '**skull**'/exp OR '**cervical vertebra**'/exp OR '**paranasal sinus**'/exp OR '**thyroid gland**'/exp OR '**head**' OR '**heads**' OR '**neck***' OR '**skull**' OR '**cranium**' OR '**cranial**' OR '**craniofacial**' OR '**mandib***' OR '**maxillary**' OR '**orbital**' OR '**paranasal**' OR '**sinonasal**' OR '**clival**' OR '**clivus**' OR '**thyroid**' OR '**laryngeal**' OR '**larynx**' OR '**salivary**')

Web of Science:

TS=(Pediatrics OR Child OR pediatric OR paediatric OR child OR adolescen OR teen OR infant)

AND TS=(Neuronavigation OR Surgical Navigation Systems OR intraoperative navigat OR neuronavigat OR surgical navigat OR stereotactic navigat OR stealthstation OR CranialMap OR NAVi OR Kick OR Stryker OR BrainLAB OR Medtronic)

AND TS=(Neoplasms OR cancer OR tumor OR tumour OR osteosarcoma OR rhabdomyosarcoma OR sarcoma OR lymphoma OR carcinoma OR histiocytosis OR Langerhans)

AND TS=(Head OR Neck OR Otolaryngology OR Larynx OR Salivary Glands OR Skull OR Cervical Vertebrae OR Paranasal Sinuses OR Thyroid Gland OR head OR heads OR neck OR skull OR cranium OR cranial OR Craniofacial OR mandib OR maxillary OR orbital OR paranasal OR sinonasal OR clival OR clivus OR thyroid OR laryngeal OR larynx OR salivary)
